# Acknowledgement and use of advance care directives and goals of care by emergency department staff: a mixed method post intervention study

**DOI:** 10.1186/s12904-024-01566-5

**Published:** 2024-10-01

**Authors:** Abdi D. Osman, Jocelyn Howell, Michael Yeoh, Louisa Lam, Daryl Jones, George Braitberg

**Affiliations:** 1 Present Address: Department of Critical Care. Parkville, University of Melbourne, Melbourne, VIC Australia; 2 Emergency Department. Heidelberg, Austin Health, Melbourne, VIC Australia; 3 College of Sports, Health and Engineering, Victoria University, University Blvd, St Albans, Melbourne, VIC VIC 3021 Australia; 4 Faculty of Health Sciences, Australian Catholic University, Melbourne, VIC Australia; 5 Intensive Care Unit. Heidelberg, Austin Health, Melbourne, VIC Australia

**Keywords:** Advance Care Planning, Emergency Department, Advance care directives, Goals of care, Medical treatment decision maker

## Abstract

**Introduction:**

Advance Care Planning (ACP) refers to a process that includes Advance Care Directives (ACD) and Goals of Care (GOC), a practice widely used for over three decades. Following the findings of an audit and a cross-sectional study in 2019 and 2021 respectively, we implemented several educational and other interventional strategies aimed at enhancing staff awareness and emphasizing the importance of recognizing and documenting of ACD/GOC. The aim of this study was to evaluate the acknowledgement and use of ACD and GOC by Emergency Department (ED) staff following these interventions.

**Method:**

We used a mixed methods approach, incorporating both observational and cross-sectional designs with reflexive thematic analysis. Data extraction for the observational study took place between 1st April and 30th June 2023 focusing on a target population of randomly sampled adults aged ≥ 65 years. Demographics and other ACD and GOC related patients’ clinical data were collected. Data collection for the cross-sectional study occurred between 19th July and 13th September 2023 targeting all ED staff. Information gathered included demographics, awareness about ACD and GOC, including storage location and implementation, as well as knowledge of Medical Treatment decision Makers (MTDM), a jurisdictional term identifying a person legally appointed to make healthcare decisions on behalf of someone who lacks decision-making capacity and other Victorian State legislative requirements were collected.

**Results:**

In the observational period, 22,335 patients attended the ED and 19% (*n* = 6546) qualified for inclusion from which a sample of 308 patients were randomly extracted. We found ACD documents were noted in the medical records of 6.5% of the sample, fewer than 8% identified in our previous study. There was no correlation between ACD record availability and age (*p* = 0.054; CI ranging from − 0.065 to 7.768). The response rate for the cross-sectional survey was 12% (*n* = 340) in contrast to earlier study with 28% (*n* = 476) respondents. Staff knowledge and familiarity with ACD was 25% and GOC 45%.

**Conclusion:**

After implementing interventions in staff education and ACP awareness, we found that ACD documentation did not improve. However, GOC documentation increased in the context of heightened institutional awareness and integration into the Electronic Medical Records (EMR).

## Introduction

Advance Care Planning (ACP) is a term that encompasses Advance Care Directives (ACD) and Goals of Care (GOC) and has been in wide use for over three decades (Morrison et al. 2021; Advance Care Planning Australia 2024). These documents and processes support patient-centered care by articulating the patient’s care wishes, particularly in relation to end-of-life and resuscitative care. However, the uptake of ACP has been less than expected (Morrison et al. 2021; Hickman et al. 2023; Rosa et al. 2023). It is therefore essential to understand barriers to successful ACP documentation, implementation and acknowledgement as this is a medico-legal requirement particularly in the state of Victoria, Australia (Parliament of Victoria 2016).

We aimed to assess the use of ACD and GOC documentation at our quaternary healthcare facility. Previous studies revealed poor documentation, utilisation, and understanding of ACDs and GOC within our Emergency Department and among its clinicians (Osman et al. 2020) We also identified inconsistencies in treatment, where some patients, who had clearly expressed their wishes against invasive or advanced life support in their legally binding ACP documents, did not have those wishes respected, despite the expectation for clinicians to adhere to them (Osman et al. 2022). Our results were consistent with the literature (Buck et al. 2021; Detering et al. 2021).

After implementing a list of interventions (Staff education, changes to electronic health information system, understanding Victoria States ACP records performance (Osman et al. 2024a), validation of MTDM records (Osman et al. 2024b), we undertook a post intervention mixed methods studies and report the results. The observational study examined the availability of ACD/GOC records, its acknowledgment and implementation in clinical decision making in comparison to our previous study (Osman et al. 2020) and the cross-sectional study with reflexive thematic analysis was undertaken to assess staff knowledge, awareness and utilisation of ACP and to evaluate the impact of our interventions by comparing it with the outcome of our previous study (Osman et al. 2022).

## Methods

In response to our previous findings, we established a special interest group to review the practice at our institution, identify barriers and develop targeted interventions to incorporate known ACP records in clinical decision making.

As part of our interventions, we implemented staff education initiatives and recommended updates to the Electronic Medical Records (EMR) to better capture pertinent information. To gain a better understanding, we also conducted a validation study of our identification of Medical Treatment Decision Makers (MTDMs), also known as surrogate decision makers’ (Osman et al. 2024b) and reviewed the jurisdiction of Victoria’s Statewide Emergency Department data (Osman et al. 2024a). These previous studies informed the methodology employed in the current study.

### Study design and setting

We employed mixed methods utilising observational and cross-sectional designs (with reflexive thematic analysis). The observational design encompassed a retrospective audit of Emergency Department Cerner FirstNet^®^ (Oracle Cerner 2024) EMR at our institution. Data extraction occurred between 1st April to 30th June 2023, to ensure that the study durations were matched with the pre-study data collection period (Osman et al. 2020). All Emergency Department patients, 65 years and older, were included in the study. The cross-sectional voluntary study involved a staff survey of all Emergency Department (ED) staff based on previously validated questionnaire tool (Osman et al. 2022). Data collection took place between 19th July and 13th September 2023.

### Sampling

For the observational study, sample size was determined using the OpenEpi sample size calculator (OpenEpi n.d.), considering an assumed frequency of the outcome (presence of documented ACD) in the population of 30% (Detering et al. 2021), with a power of 80% and a confidence interval of 95%. This calculation yielded a required sample size of 308. Using STATA statistical software (StataCorp 2023), a random sampling technique was employed to select 308 patients from the pool of eligible individuals. Subsequently, data analysis was performed using the information gathered from these 308 patient records. For the cross-sectional study, convenient sampling was used where all ED staff (*n* = 340) were invited to participate in a study which was based on our previous study design with validated questionnaire.

### Observational data extraction/collection

The variables extracted from the EMR for the observational study included demographics, triage category, time of arrival at ED. Key clinical information related to ACP was also captured, such as the presence of ACD within the EMR, which was accessed through a link to the scanned medical records (SMR). Additionally, the presence of GOC, displayed in the patient identification banner within the EMR, was noted. This information is required to be completed and updated by ED clinicians for every patient presentation.

Other critical variables included the dates of any ACD/GOC noted in the EMR or the SMR documents, whether the treating clinician acknowledges the ACD in the ED discharge summary, and whether the treatment provided complied with ACD/GOC. Compliance was assessed by cross-referencing the treatment administered with the documented preferences and goals outlined in the ACD/GOC. Further details, such as the patient’s disposition destination, the admitting specialist and the number of presentations were also recorded.

To ensure the reliability of this data, blinded inter-rater reliability testing was performed. In this process, 20% of the records were randomly selected and reviewed by two senior clinicians. They were instructed to document where there has been an acknowledgement of ACD or treatment compliance with ACD/GOC records. The clinicians were to mark “Yes” if there was acknowledgement or compliance, “No” if there has been no acknowledgement or if treatment was inconsistent with the ACD/GOC, and “N/A” if no ACD/GOC records were present or if the patient’s condition or treatment was not relevant for such a review. The raters report which was in a Microsoft Office Excel^®^ was exported to STATA 18e statistical software (StataCorp 2023) where kappa-interrater agreement analysis was performed.

### Cross-sectional data collection

The questionnaire was based on a previously validated tool (Osman et al. 2022) and the variables encompassed staff demographics, awareness about ACD and GOC, including storage location and implementation, and awareness about MTDM and Victoria State’s legislative requirements. Data were collected using online survey software (REDCap). Invitation to participate was sent through the department’s electronic newsletter with a QR code and a link to the survey on the 19th of July 2023, followed by weekly reminders in the newsletter until 13th September 2023.

### Ethics

Ethics approval for the study was obtained from the Austin Health Human Research Ethics committee (HREC/69061/Austin/2020). Patient consent was waived for the observational component of the study as the data was de-identified.  For the cross-sectional study, the participants information sheet, consent form and survey were all administered electronically using REDCap. Participants were provided with the information sheet and asked to provide consent by selecting "yes" in response to the question, "Do you agree to participate in the survey?" Upon consent, the survey became accessible. If they selected "no," the survey page closed, displaying a "Thank you for visiting" message.

### Observational data analysis

Numerical data for both studies were presented as counts and percentages. In cases where there were missing values the data were presented as n (number of cases) / N (number of instances where the value was known) with no assumptions made about missing data. Age was the only continuous variable which was normally distributed (skewness of 0.207 and Kurtosis of 0), and the rest of the variables were categorical. Chi-square or Fisher’s exact test, T-test, regression, and multiple regression tests were undertaken as appropriate. Statistical significance was indicated by a two-sided p value < 0.05 and Confidence Interval.

### Cross-sectional data analysis

The questionnaire responses were exported to STATA 18e statistical software (StataCorp 2023) for analysis. Following exportation to STATA, the open-ended responses were retrieved from the software’s data editor and transcribed into a Microsoft Office Excel^®^ spreadsheet. Using a reflexive thematic analysis approach (Braun and Clarke 2021; Byrne 2022), after familiarization with the data, each response was grouped into a theme. Upon completion of grouping by the lead author, it was reviewed by the team. In cases where there were discrepancies in groups/themes, the group reached consensus moderated by the chief investigator on which themes to include considering the study aim.

## Results

### The observational study

In the observational study, among the 22,335 patients who attended the ED during the study period, 19% (*n* = 6546) were selected for random sampling, resulting in a sample of 308 patients. The sample characteristics and their association with ACD presence (using chi square/Fisher’s exact test) are shown in Table 1 incorporating previous study findings (Osman et al. 2020).

**Table 1:** Participant characteristics of current study (*n* = 308) and association with ACD presence

		2019 study (*n* = 300)	Current study (*n* = 308)	
Variable	Type	Frequency n (%)	Frequency n (%)	*p* value
Gender	Female	152 (50.7%)	163 (52.9%)	*p* = 0.847
	Male	148 (49.3%)	145 (47.1%)	
Age categories	≤ 74 years	106 (35.3%)	116 (37.7%)	*p* = 0.136
	75–84 years	122 (40.7%)	94 (30.5%)	
	≥ 85 years	72 (24.0%)	98 (31.8%)	
Triage categories	1- Resuscitation	2 (0.7)	1 (0.3%)	*p* = 0.001
	2- Emergent	48 (16.0%)	90 (29.2%)	
	3- Urgent	141 (47.0%)	149 (48.3%)	
	4- Semi-urgent	102 (34.0%)	59 (19.2%)	
	5- Non-urgent	7 (2.3%)	9 (2.9%)	
ACD present	Yes	24 (8.0%)	20 (6.5%)	
GOC^a^ Present	Yes	112 (37.3%)	149 (48.4%)	*p* = 0.003
Acknowledgement of ACD^b^/GOC presence?	Yes	11 (9.5%)	8 (2.6%)	*p* < 0.001
	No		135 (43.8%)	
	N/A		165 (53.6%)	
Treatment consistency with ACD	Yes	116 (38.6%)^c^	20 (6.5%)	*p* < 0.001
	N/A		288 (93.5%)	
Treatment consistency with GOC	Yes		140 (45.5%)	*p* = 0.001
	N/A		168 (54.5%)	
Disposition destination	Home	148 (49.3%)^d^	146 (47.4%)	*p* < 0.001
	General Ward	134 (44.7%)	136 (44.2%)	
	Aged care facility	12 (4.0%)	11 (3.6%)	
	Did not wait		10 (3.2%)	
	ICU/Theatre	6 (2.0%)	5 (1.6%)	
No. of presentations	Minimum		1	*p* = 0.013
	Average		3	
	Maximum		23	

^a^ *GOC* Goals of Care

^b^ *ACD* Advance Care Directive

^c^This included N/A response and combined with GOC

^d^Includes Did Not Wait

The age range of the study group was 65 to 100 years, with a mean age of 78 years. When correlating the availability of ACD records against age, no statistically significant outcome was observed (*p* = 0.054; CI -0.065 to 7.768), implying that ageing has no association with ACD record availability over the age of 65. However, there was a significant association between age and GOC documentation availability (*p* < 0.001; CI = 3.024 to 6.752), suggesting an increased availability of GOC records with advancing age.

The gender distribution among the group was nearly equal and revealed no statistically significant relationship with the presence of ACD or GOC records (*p* = 0.848; CI = -0.250 to 0.206 or *p* = 0.794; CI = -0.127 to 0.097 respectively).

Among the available ACD records (*n* = 20), 50% dated back to between 2010 and 2019, with the other 50% recorded between 2020 and 2023. 96% of GOC documents (*n* = 149) were recorded on or after 2020, with most occurring after 2022 when the workflow was integrated into the EMR. Out of 169 participants who had ACD/GOC records available, *n* = 143 had complete clinical discharge documentation and of this, 5% had an acknowledgement of ACD/GOC records with an interrater reliability of 0.83. Treatment consistency with ACD were at 100% for the 20 ACD records available (interrater reliability of 0.97) and GOC were also at 100% (*n* = 140) with an interrater reliability of kappa 0.90.

The patient’s discharge destination from ED (Table 1) was mainly either to home (*n* = 146, 47%) or admission to the general ward (*n* = 136, 44%). There was a significant association between discharge destination and the availability of both ACD and GOC records (*p* = 0.007; CI = 0.351 to 2.142 and *p* < 0.001; CI = 0.981 to 1.818 respectively) which indicated a tendency of document availability for admitted patients ACD (65%) and GOC (63%).

### The cross-sectional study

Direct comparison of the outcomes has been made impossible by significant proportional differences of participants populations: *n* = 134 (28%) in the pre-study compared to *n* = 40 (12%) in the post study.

#### Participants demographics

Forty staff members (12%) responded to our invitation to participate. The respondents’ demographics details are shown in Table 2.

**Table 2:** Staff demographics and ACP knowledge and awareness

Variable	Type	Frequency *n* (%)
Gender	Female	26 (65%)
	Male	13 (33%)
Age ranges	20-25yrs	3 (7%)
	26-35yrs	11 (28%)
	36-45yrs	13 (32%)
	46-55yrs	7 (18%)
	≥ 56yrs	6 (15%)
Discipline	Admin	7 (18%)
	Nursing	16 (40%)
	Medicine	14 (35%
	Care coordinators	3 (7%)
Duration of practice in facility	Min	1yr
	Average	4yrs
	max	5yrs
ACP Education participation	Yes	8 (20%)
Familiarity with ACD documents	Very familiar	10 (25%)
	Somewhat familiar	26 (65%)
Familiarity with GOC documents	Very familiar	18 (45%)
	Somewhat familiar	22 (55%)
Knowledge of where to find existing ACD records	Yes	28 (70%)
Knowledge on where to find existing GOC records	Yes	32 (80%)
Easy to locate existing ACD/GOC record?	Yes	20 (50%)
How well the system captures ≥ 65yrs ACD and GOC Records	Well	6 (15%)
	Fair	11 (27%)
Knowledge on Victorian Medical Treatment Planning & Decisions ACT 2016 legislation?	Very well	1
	Well	6 (15%)

For ACD education participation, among the eight (20%) who reported having participated, further enquiries on when they undertook the study produced the results below.

*“Unsure – Institutional ACP department conducted it a few years ago”*

“long time ago*”*

“2022*”*

“many years ago*”*

“Approx 4 years ago when Advanced care planning was changed*”*

“Unsure of date but not recently*”*

“When the organisation did its first assisted dying*”*

“Can’t remember*”*

Most of the participants (*n* = 28, 70%) were aware of where to find existing ACD records. Upon further enquiry, these respondents mentioned the correct location of the said document. Similarly, regarding GOC, the respondents who said they were aware of where to find the records (*n* = 32, 80%) provided the correct response when asked about the exact location. Unlike ACD, GOC documents are generated in the ED, which can result in multiple storage points.

When enquiring about how effectively the system captures ACD and GOC records for patients aged ≥ 65 years, most of the respondents (53%) indicated poorly or very poorly. This response was followed by participants reporting that they did not read the organisational guidelines on GOC (82%), revealing a statistically significant association between those respondents who believed that the system did not adequately capture ACP information and those that indicated they had not read the guidelines (*p* = 0.001; CI = 1.081 to 3.797).

Staff knowledge regarding the Victorian Medical Treatment Planning & Decisions ACT (VMTPDA) 2016 legislation was predominantly categorised as poor or fair (38% for each). Only seven staff members reported understanding it well or very well, and all seven gained this knowledge primarily through self-learning. Better awareness and comprehension of the VTMPDA 2016 was found to have a statistically significant correlation with attending an ACP education session (*p* < 0.001; CI = 1.917 to 4.644) or being familiar with the organisational guideline on GOC (*p* = 0.001; CI = 0.976 to 3.671).

Staff were offered the opportunity to respond to open ended questions regarding how to improve the electronic storage and access of ACD and GOC documentation (*n* = 31 with meaningful responses), the implementation of existing ACD and GOC records (*n* = 29), and the streamlining of consent for patients with cognitive impairment and no existing ACD records (*n* = 29). Using a reflexive thematic approach, the themes reported in Table 3 emerged.

**Table 3:** Thematic analysis of staff response to enquiries on ACD and GOC storage, implementation, and consenting process

Question	Theme (frequency)	Theme Group	Related quote
How can we improve the ACD and GOC documentation electronic storage location and access?	Connecting alert/Icon in EMR front page (5)	C002	*“I think it’s now on first net? But still not sure. Would be great if there was a first net icon that could link you straight to the last GOC discussion. I usually spend some time clinking around in SMR until I find it.”*
	Dedicate section in main EMR (15)	D015	*“Needs a dedicated section in EMR to locate ACD and GoC documentation all together, and ensure the most recent is accessed. Some way to have detailed ACD plans uploaded to EMR to capture exact wording on ACD, if some info is on Cerner and some on SMR this can be confusing for which one to look at.”*
	Recurrent update every admission (9)	R004	*“Make sure that firstnet keeps records up to date, and that doctors, both ED and inpatient, are making sure to document goals of care at the earliest opportune moment. Often it seems ED doctors disregard this, waiting for inpatient teams, and it creates issues for nursing staff”*
	Educate staff on completing/filing (2)	E002	*“Education sessions regarding where to find documentation, who can make decisions, who can speak with patient around decisions”*
**QuestionTheme (frequency) Theme groupRelated quote**			
How can we improve the implementation of existing ACDs and GCCs?	Pop-up trigger notification (13)	PT003	*“Can FirstNet/CERNER have a trigger built into it to alert when someone does not have a GOC documented? I continue to be astounded by the frequency with which mainly haem and onc patients present to ED in extremis; they are suffering from an incurable illness for which they are receiving palliative / symptom directed management and NOONE in the home treating team has ever had a GOC discussion with the person and/or significant others. Surely for this particular subset of clients and others with chronic incurable illness*,* a GOC discussion should be mandatory?”*
	Staff Education (5)	SE003	*“Often they (GOC) are written in the medical / surgical admission notes but may not be verbally communicated or updated in First net so you don’t realise until you are handing over. So better documentation, maybe decisions discussed with patients earlier by ED team. Written ACD brought in by ambulance may not get updated onto our GOC so can be confusing, pretty sure to follow the written instructions, but would be good to have it documented by our team.”*
	Clarity and consistency of records (11)	CC001	*“Clarity on the interventions that are offered and should not be considered needs to be more explicit and simpler. Carry forward GoC on one admission automatically to the next.”*
**Question Theme (frequency) Theme group Related quote**			
How can we streamline obtaining consent for patients with cognitive impairment and with no ACD?	System or staff guide (3)	SG001	*“have a section for it that is more visible and easier to find in either ED Summary or MTDM/Patient information on FirstNet that stays there from previous admissions - as the alert goes away after each admission”*
	MTDM contact maintained (11)	MTD009	*“I’m not sure except to suggest that directives are in place on entering any facility. I understand not everyone has an enduring power of attorney. Perhaps there needs to be hospital policy of how best to care for a patient by considering best outcome for patient”*
	Unsure (14)	U004	*unsure. I think we need to get better at our cognitively intact patients before we try harder tasks.*

## Discussion

Advance Care Planning, which was first established more than three decades ago (Morrison et al. 2021) is still full of challenges (Lund et al. 2015; Sinclair et al. 2023). While ACP’s aim is well defined in relation to people planning for their future healthcare (Advance Care Planning Australia 2024), accurate documentation of ACP has proven to be a challenge. The advent of EMR was hoped to ameliorate this challenge, but this has not been borne out in practice (Joshua et al. 2021). Given the potentially overwhelming list of challenges, some in the literature recommend a rethink of its main purpose and outcome (Chetna et al. 2022).

Our studies, both before and after intervention, highlight the challenges surrounding the availability, access and implementation of ACP. The pre studies (Osman et al. 2020, 2022) have shown deficiencies in record availability, access, implementation and staff knowledge and awareness. During the intervention, we also undertook studies to evaluate our MTDM identification processes (Osman et al. 2024b) as well as our state EDs’ performance based on records kept by the state department of health (Osman et al. 2024a) which also pointed to deficits in the overall ACP processes.

The observational post study revealed 6.5% of the target population had an ACD record available, which is below the availability of 8% for a similar population in our 2019 pre study, GOC record availability and use in the post study was better, indicating 48% in comparison to 37% in the pre study (Osman et al. 2020). While the improvement in GOC documentation can be attributed to integration of GOC with EMR in 2022 and institutional impetus to improve completed GOC records, as supported by literature (Curtis et al. 2023), there is no likely reason for the decline in ACD records presence given the expectations of a rise after the COVID-19 pandemic. We suggest further study to determine if this reduction is random or reflects a larger trend.

Out of the available ACD and GOC records, in 143 instances where there was an available discharge summary, only eight (5%) of these summaries recorded or acknowledged the ACP which demonstrates poor ACD uptake by the community, GOC documentation and their implementation (Buck et al. 2021; Detering et al. 2021). Independent reviews of the ACD/GOC acknowledgement and treatment consistencies indicated a high Cohen’s kappa interrater reliability as reported. It is likely that clinicians may have been aware of the intent displayed in ACP documents and acted accordingly, even if this was not explicitly described in the medical notes (Houben et al. 2014).

The cross-sectional post intervention survey revealed improved familiarity level of ACD by staff at 25%. This is slightly better than our pre study performance at 19%, but GOC was slightly reduced at 45% now versus 49% in the pre study (Osman et al. 2022). One important difference between the pre and post studies was the response rate at 134 of 476 target population (28%) for the pre study versus 40 of 340 target population (12%) for the post study. We hypothesise that “survey fatigue” may have contributed to this, as our survey was conducted at the same time as several others in the ED.

The variable response to ACP implementation in relation to ACP knowledge in this study mirrors the findings of the pre study (Osman et al. 2022), despite differences in participant population density. Staff recommendations were similarly on staff education and system wide improvement, which is supported by the literature (Hickman et al. 2023; Krotova et al. 2023). Based on our experience, staff education is challenging with high staff turnover, so rather than one-off education sessions, ACP educational programs should be part of annual educational calendars of clinical facilities.

### Limitations

We did not undertake comprehensive records review but rather a small sample of the records. Our cross-sectional survey had a low response rate thereby making it susceptible to selection bias. In addition, the inability to verify whether the survey responders have participated in the educational intervention implemented in the unit is an important limitation. Interventions happening during COVID-19 peak seasons, thereby interrupting our intervention plans and resulting in higher than usual staff turnover. Single coder grouping themes has the potential for bias in reporting. The study took place in a single site and together with the low staff response to the post intervention survey potentially limits external validity. The absence of ACD/GOC documents for a significant portion of participants may weaken the study’s statistical power.

## Conclusions

After implementing interventions in staff education and ACP awareness, we found that ACD documentation did not improve. However, GOC documentation increased in the context of heightened institutional awareness and integration into the EMR.

## Data Availability

Data will be availed on reasonable request.
